# Effects and contextual factors of a diet and resistance exercise intervention vary across settings: an overview of three successive ProMuscle interventions

**DOI:** 10.1186/s12877-021-02733-6

**Published:** 2022-03-09

**Authors:** Berber G. Dorhout, Lisette C.P.G.M. de Groot, Ellen J.I. van Dongen, Esmée L. Doets, Annemien Haveman-Nies

**Affiliations:** 1 Division of Human Nutrition and Health, Wageningen University and Research, Stippeneng 4, PO Box 17, 6700 AA Wageningen, the Netherlands; 2 Food, Health and Consumer Research, Wageningen Food and Biobased Research, Wageningen, the Netherlands; 3 Chair group Consumption and Healthy Lifestyles, Wageningen University and Research, Wageningen, The Netherlands; 4 GGD Noord- en Oost-Gelderland, Academic Collaborative Center AGORA, Zutphen, the Netherlands

**Keywords:** Lifestyle, Translation, Context, Implementation

## Abstract

**Background:**

Although many effective interventions have been developed, limited interventions have successfully been implemented. An intervention that was translated across settings is ProMuscle: a diet and resistance exercise intervention for older adults. However, varying contexts often lead to varying effects due to contextual factors (characteristics of individuals, organizations, communities or society). The current study aimed to gain insights into effects and contextual factors of ProMuscle in the controlled setting (ProMuscle: PM), real-life setting (ProMuscle in Practice: PiP), and real-life setting of the implementation pilots (ProMuscle Implementation Pilots: IP).

**Methods:**

Data from the intervention arms of PM (N = 31) and PiP (N = 82), and from IP (N = 35) were used. Physical functioning (chair-rise test) and leg strength (1-10 repetition maximum) were measured at baseline and after 12-weeks intervention. Paired t-tests and General Linear Models were used to study changes after 12 weeks and differences between interventions. To explore contextual factors, researchers of PM and physiotherapists and dietitians of PiP and IP were interviewed. Factors were categorized according to the five domains and its underlying constructs of the Consolidated Framework for Implementation Research (CFIR).

**Results:**

Improvements on chair-rise performance were found in PM (-2.0 ± 7.0 s, p = 0.186), PiP (-0.8 ± 2.9 s, p = 0.019) and IP (-3.3 ± 4.2 s, p = 0.001). Similar results were found for leg strength in PM (32.6 ± 24.8 kg, p < 0.001), PiP (17.0 ± 23.2 kg, p < 0.001), and IP (47.8 ± 46.8 kg, p < 0.001). Contextual factors that contribute to explaining the relatively high effects in IP included room for adapting and tailoring the intervention, involvement of experienced professionals, availability of and access to facilities, and participants characteristics.

**Conclusions:**

Effects of the intervention appeared to be strongest in the real-life setting of the implementation pilots. Specific contextual factors contributed to explaining the different findings across settings. Future studies should investigate crucial factors that determine successful implementation of interventions in the real-life setting, to ensure that effective interventions are put into action and reach a broad population.

**Trial registration:**

The ProMuscle intervention was registered in the Trial Registration (clinicaltrials.gov identifier: NCT01110369) on February 12th, 2010. The ProMuscle in Practice intervention was registered in the Netherlands Trial Register (NTR6038) on August 30th, 2016. Trial registration was not needed for the ProMuscle Implementation Pilots as this research did not fall within the remit of the Dutch ‘Medical Research Involving Human Subjects Act’.

## Introduction

Although many lifestyle interventions are being developed and achieve promising effects in clinical settings, only few interventions are in the end successfully implemented and disseminated in the real-life setting [1]. In fact, a large part of research is never translated into practice [2]. The process of transforming basic research into a widely implemented intervention is often complex, time consuming, and expensive [3, 4].

As a large part of effective treatments and interventions is not yet available to a wide population, it is essential to study implementation of health promotion interventions [1, 5]. This is particularly of great importance for older adults, as the ageing population is expected to grow even more in the coming decades [6, 7]. Health promotion programs can contribute to the prevention of the negative consequences of ageing, such as development of diseases or decline in functioning [5]. An intervention that aimed to counteract functional decline in older adults is the ProMuscle program, consisting of resistance exercise and protein supplementation [8]. During the past decade, the program has moved through a translation process from basic and efficacy research towards implementation. Starting with testing the efficacy of the combination of nutrition and exercise in improving muscle health (ProMuscle) [8, 9], followed by designing and evaluating an intervention in the real-life setting (ProMuscle in Practice) [10–12], and currently exploring the possibilities of implementing the intervention in multiple organisations and populations (ProMuscle Implementation Pilots). The basic elements, progressive resistance exercise and increased protein intake, were retained in every intervention. However, the adaptable content of the intervention, the role of involved professionals, and the influence of other contextual factors impacting the intervention effects are expected to vary across settings.

It is common to find effects fading away or differing across settings, especially when an intervention is implemented in the real-life setting [13]. The varying effects can be due to the influence of context in the different settings [14]. Context can be defined as “a set of characteristics and circumstances that consist of active and unique factors, within which the implementation is embedded” [14]. The Consolidated Framework For Implementation Research (CFIR) can be used to investigate which form of intervention works where and why across various settings [15]. CFIR includes five major domains: intervention characteristics, outer setting, inner setting, characteristics of the individuals involved, and the process of implementation. These domains are subdivided into constructs. For example, the domain ‘inner setting’, referring to the organisation in which the intervention is being conducted, can be subdivided into the constructs: structural characteristics of the organisation, networks and communications, culture, implementation climate, and readiness for implementation. Different sets of constructs interact with interventions when conducting the program in a controlled setting compared to implementing the program in a practice setting. When taking the inner setting as an example, structural characteristics of an organisation may play a role in the controlled setting, whereas readiness for implementation is expected to be more important in the practice setting. The CFIR domains and their constructs can help unravel which factors play a role in a specific setting and can help explain intervention effects in the different settings [15].

Therefore, the aim of this study was to explore contextual factors that can help to explain the potential differences in effects of the three successive ProMuscle interventions across settings.

## Methods

This paper includes data from three versions of the ProMuscle intervention: ProMuscle (clinical setting), ProMuscle in Practice (real-life setting), and the ProMuscle Implementation Pilots (real-life setting of the implementation pilots). An extensive description of the study design, study population, and intervention of ProMuscle and ProMuscle in Practice can be found elsewhere [8, 10]. In short, methods for each program are described below.

### Study design and setting

*ProMuscle (PM)* was a randomized, double-blind, placebo-controlled trial with 2 arms in parallel. Participants were randomly allocated to the intervention or control group, stratified by sex. Both groups were included in a 24-week resistance exercise program. The intervention group received protein supplementation, whereas the control group received placebo supplementation. The intervention was delivered by researchers at a university in the Netherlands, in a room equipped as gym location.

*ProMuscle in Practice (PiP)* was a randomized controlled multicentre intervention study, implemented at five Dutch municipalities. Participants were randomly allocated to the intervention or control group, stratified by sex and frailty state. The program focused on resistance exercise and increasing dietary protein intake, implemented by physiotherapists and dietitians. Participants of the intervention group started with an intensive support intervention (week 1-12), followed by a moderate support program (week 13-24). The control group received no intervention.

*ProMuscle Implementation Pilots (IP)* were two case studies, including only an intervention group (pre-test post-test). The IP were implemented by physiotherapists and dietitians at two separate physiotherapist and dietitian practices in two Dutch municipalities. The 12-week program included progressive resistance exercise and a nutrition program, consisting of individual consultations and group meetings.

In the current paper, we included the intervention groups of the first 12-week intervention period of PM, PiP, and IP.

### Study population

*ProMuscle* – Older adults were recruited from an existing database, via distribution of flyers and by organising local information meetings. PM included older adults (≥65 years) who were prefrail or frail according to the Fried criteria [16], after checking medical history and exclusion criteria (described in detail elsewhere [8]). The Wageningen University Medical Ethical Committee approved the study and participants gave their written informed consent.

*ProMuscle in Practice* – Older adults were recruited mainly through local media. PiP included older adults (≥65 years) being prefrail or frail according to the Fried criteria [16], or being non frail but experiencing difficulties in daily activities and being inactive (defined as not participating in resistance exercise >30 min a day on more than 2 days a week). Exclusion criteria were checked by the older adults’ general practitioner (GP), including renal functioning (eGFR) (described in detail elsewhere [12]). The study protocol was approved by the Wageningen University Medical Ethics Committee and included participants provided written informed consent before participation.

*ProMuscle Implementation Pilots –* Older adults were recruited through local media, and word of mouth by the physiotherapist, or were referred by the practice assistant of the GP. The IP included older adults (≥65 years) who were either deemed suitable by the physiotherapist or who were referred by the practice assistant of the GP (with one of the following reasons: improving muscle strength; insight in and improving intake of protein; recovery after inactive period). Medical status and renal functioning (eGFR) were checked in collaboration with the GP, before starting the intervention. No medical ethical approval was needed as this research did not fall within the remit of the Dutch ‘Medical Research Involving Human Subjects Act’ (in Dutch: WMO). Participants provided informed consent before participation.

### Intervention

The content of the PM, PiP and IP interventions is described in Tables 1 and 2, subdivided into the exercise program (Table 1) and the nutrition program (Table 2). In short, the basic elements of the intervention, providing RE training sessions and increasing dietary protein intake, were present in each intervention. Differences across exercise programs are related to location, type of guidance, and structure of the training sessions. Differences across the nutrition programs are related to type and frequency of guidance and protein product.

**Table 1:** Intervention description: Content of exercise program (12 weeks) in the various ProMuscle interventions

	**ProMuscle**	**ProMuscle in Practice**	**ProMuscle Implementation Pilots**
Setting	Training at university in room equipped as gym location for the trial.	Training at local care organization in room equipped as gym location for the trial (in neighbourhood of older adults).	Training at training room of the local physiotherapist practice (in neighbourhood of older adults).
Frequency	Two times per week for one hour.	Two times per week for one hour.	Two times per week for one hour.
Intake	No intake consultation. Medical assessment was performed during inclusion.	No intake consultation. Before the start of the program, physiotherapists received participants’ medical and baseline strength-related measures from researchers.	Individual intake consultation with physiotherapist, to assess training level, potential injuries, and medical status.
Supervision	Researcher assisted by trained students. One trainer per two participants. 5-6 participants per group.	Physiotherapists and their assistants. Two to three trainers per group. 4-7 participants per group.	Physiotherapists and their assistants. One trainer per group. First 2-3 weeks: two trainers. 5-8 participants per group.
Type of guidance	Researcher organized individual training schedules; individual guidance during exercise performance.	Physiotherapists organized training sessions according to detailed training protocol. Tailored the training intensity when necessary (e.g. in case of an injury).	Physiotherapists used the protocol as a guideline for creating a training schedule and tailored the training intensity to individuals’ capabilities and limitations.
Training session structure	Warming-up; progressive resistance exercises on leg press, leg extension, chest press, lat pulldown, pec deck, and vertical row machines; warm-down. Focus on main muscle groups.	Warming-up; progressive resistance exercises on leg press, leg extension, chest press, lat pulldown, and vertical row; warm-down. Focus on main muscle groups.	Dependent on training location, including: Warming-up; progressive resistance exercises on leg press, leg extension, back extension, pull down, cable row, chest press; warm-down. Focus on main muscle groups. Tailored to individual needs: cardio exercises (exercise bike, treadmill, cross trainer), squats with free weights.
Workload	Started at 3-4 sets of 10-15 repetitions (50% of 1-RM) and increased toward 3-4 sets of 8-10 repetitions (75% of 1-RM).	Leg exercises: started with 3-4 sets of 15 repetitions (50% of 1-RM) and increased toward 4 sets of 8 to 12 repetitions (75%-80% of 1-RM). Other exercises: According to insights of physiotherapist. Adjustments made e.g., in case of injury.	Leg exercises: Started with 3-4 sets of 15 repetitions (50% of 1-RM) and increased toward 4 sets of 8 to 12 repetitions (75%-80% of 1-RM) (location 1). Building up intensity according to own insights and based on 3-RM measurement on leg press and leg extension, intensity was on average increased with 5% per weight increase (location 2). Other exercises: According to insights of physiotherapist. Adjustments made e.g., in case of injury.

**Table 2:** Intervention description: Content of nutrition program (12 weeks) in the various ProMuscle interventions

	ProMuscle	ProMuscle in Practice	ProMuscle Implementation Pilots
Type and frequency of guidance	Short explanation on protein drinks at start of intervention by research dietitian (no consultation).	Individual consultations with dietitian; before intervention, after 6 weeks, and additional phone consultation when needed.	Individual consultations with dietitian; at the start of the intervention, in week 2 or 3, and at the end of the intervention and additional consultations when needed. One group meeting at the end (location 1). One individual consultation with dietitian at the start, three group meetings (location 2).
Type and frequency of protein product	Provision of 250mL protein supplemented beverage containing 15 g protein. One drink directly after breakfast, one drink directly after lunch.	Provision of range of free protein-rich products, such as dairy drinks, cheese, or yoghurt. Tailored to individual needs and preferences. Protein-rich products were mainly consumed during breakfast and lunch, aimed at reaching consumption of 25g protein per main meal.	No provision of supplements or products. Advise was focused on animal-based as well as plant-based protein. Tailored to individual needs and preferences. Aimed at 20-25g protein per main meal.

### Quantitative measures

#### Baseline characteristics

Questionnaires were used to collect baseline characteristics including age and sex. Body weight was measured to the nearest 0.1 kg using a digital scale, and height was measured to the nearest 0.1 cm using a stadiometer.

For this study, we included measures that were collected in all three intervention groups at baseline and after 12 weeks of intervention: chair-rise test and leg muscle strength.

#### Chair-rise test

Physical performance was measured by the chair-rise test [17]. The measurement was performed according to a standardized protocol. In PM and PiP, measurements were conducted by trained researchers and their assistants. In IP, measurements were conducted by researcher-instructed physiotherapists.

#### Leg muscle strength

In PM, researchers performed 1 Repetition Maximum (1-RM) strength tests on leg press machines. In PiP, researchers measured muscle strength through 3-RM tests at leg press machines. In both PM and PiP, measurements were performed according to a standardized protocol. In IP, physiotherapists measured muscle strength through 3-RM at leg press machines according to protocol. In some case in PiP, and more often in IP, more repetitions were used, if necessary (up to 10-RM), to align with older adults’ physical capacities. The RM scores were recalculated to 1-RM, based on the formula of Brzycki [18].

### Qualitative measures

Semi-structured interviews were conducted to collect information regarding process indicators and in particular contextual factors that could influence intervention outcomes and intervention implementation. A PhD-level researcher and a MSc-level researcher conducted a face-to-face semi-structured interview with the main researcher and a research-assistant who delivered PM [11]. A MSc-level researcher conducted semi-structured interviews via telephone with 18 physiotherapists and 8 dietitians involved in the first 12 weeks of PiP [19]. Interview questions were based on pretested interview guides [11]. A MSc-level researcher conducted semi-structured interviews via video calling with two physiotherapists and one dietitian involved in IP. Interview questions were based on interview guides from PiP and were supplemented with questions on contextual factors.

### Statistical analyses

Univariate procedures were used to check for normal distribution of the data. Baseline data were expressed as means with standard deviations or as percentages. Baseline differences between treatment groups were analysed using one-way ANOVA for continuous data, and Pearson’s chi-squared tests for categorical data. Paired samples t-tests were used to analyse changes between the pre-test and post-test measurements (baseline vs. week 12) for each of the interventions separately. Differences in effects between the three interventions were analysed using General Linear Models. Separate models were conducted for the chair rise test and leg press strength. The dependent variable included the changes after 12 weeks (calculated by subtracting pre-test measurement from post-test measurement). Program (PM, PiP, IP) was included as independent variable. Multiple comparisons (post-hoc tests) were conducted to study the differences in effects between the programs. The model was adjusted for sex to study the influence of sex on differences in effects across settings. The results of the adjustment are highlighted in the discussion section. Data of complete cases (measurement at baseline and week 12) were included. Data was analysed using SPSS version 23 (IBM Corp., Armonk, NY). Statistical significance was indicated with p-value<0.05.

Qualitative data were analysed in Atlas.ti, version 9. Interviews were taped, transcribed verbatim, and transcripts from the interviews were analysed and coded. Results were classified according to relevant constructs of the five domains of CFIR. Constructs that were highlighted in the interviews were selected and included in the classification [15].

## Results

### Baseline characteristics

Data were normally distributed. Table 3 presents the baseline characteristics of participants for each program separately. No baseline differences were found between the three settings, except for sex. PiP and IP comprised more female participants compared to PM.

**Table 3:** Baseline characteristics﻿ of intervention group participants from ProMuscle (PM), ProMuscle in Practice (PiP), and the ProMuscle Implementation Pilots (IP)

	ProMuscle	ProMuscle in Practice	ProMuscle Implementation Pilots
	N=31	N=82	N=35
Age (years)	77.7 ± 8.8	74.7 ± 5.8	75.0 ± 6.5
Sex (n female, %)	11 (36%)	51 (62%)	28 (80%)
Bodyweight (kg)	79.5 ± 15.8	76.1 ± 14.4	75.4 ± 12.8^a^
Height (m)	1.67 ± 0.1^a^	1.68 ± 0.1	1.67 ± 0.1^b^
BMI (kg/m^2^)	28.6 ± 4.6^a^	27.1 ± 4.8	27.3 ± 3.9^b^

Note: Data is presented as means ± SD or n (%). BMI = Body Mass Index

^a^N=30; ^b^N=22

### Effects in ProMuscle, ProMuscle in Practice, and the ProMuscle Implementation Pilots

**Fig. 1:**
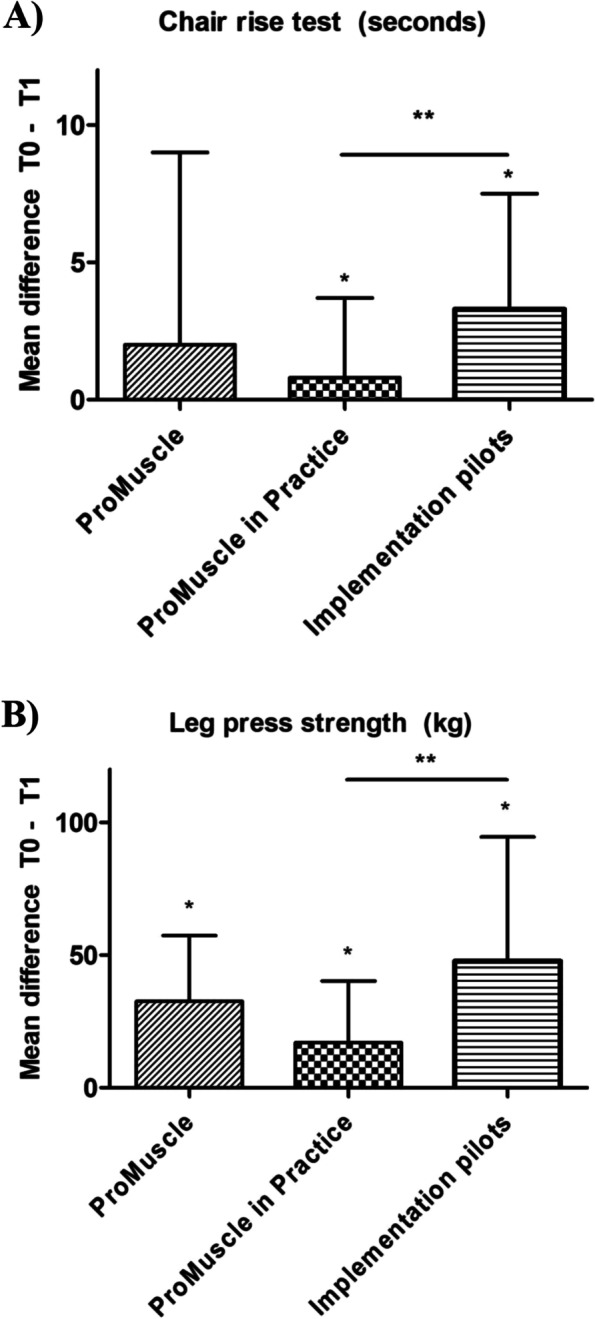
Effects after 12 weeks in chair-rise performance (**A**) and leg press strength (**B**) for each of the interventions separately. *Statistically significant effect after 12 weeks (p < 0.05). **Statistically significant difference between two interventions (p < 0.05)

The effects on chair-rise test (seconds) and leg press strength (kg) were investigated in the total study population and in the intervention group of each program separately (Table 4, Fig. 1). No baseline differences on chair-rise test and leg press strength were found between the three settings. Results of GLM show a significant effect of version of the ProMuscle program on the effects in chair-rise (F_2, 120)_ = 3.5, p = 0.035) and leg strength (F_2, 122)_ = 10.6, p < 0.001). Table 5 shows the results of the post-hoc tests of GLM, comparing the effects between PM and PiP, PM and IP, and IP and PiP.

**Table 4:** Results of chair-rise test (seconds) and leg press strength (kg) after 12 weeks in the intervention group of ProMuscle, ProMuscle in Practice and the ProMuscle Implementation Pilots

**Chair-rise test (sec)**	**Complete cases**	**Wk 0** **Mean ± SD**	**Wk 12** **Mean ± SD**	**Mean difference** **± SD**	**p-value**
Total	N=121	14.5 ± 4.9	13.0 ± 4.4	-1.6 ± 4.3	0.001
ProMuscle	N=23	16.2 ± 7.6	14.2 ± 6.3	-2.0 ± 7.0	0.186
ProMuscle in Practice	N=73	13.8 ± 3.4	13.0 ± 3.4	-0.8 ± 2.9	0.019
ProMuscle Implementation Pilots	N=25	14.8 ± 5.2	11.5 ± 4.9	-3.3 ± 4.2	0.001
**Leg press strength (kg)**	**Complete cases**	**Wk 0** **Mean ± SD**	**Wk 12** **Mean ± SD**	**Mean difference** **± SD**	**p-value**
Total	N=123	129.1 ± 34.7	157.7 ± 45.7	28.6 ± 34.0	0.001
ProMuscle	N=26	127.3 ± 29.2	159.9 ± 38.8	32.6 ± 24.8	0.001
ProMuscle in Practice	N=64	134.6 ± 38.3	151.6 ± 40.3	17.0 ± 23.2	0.001
ProMuscle Implementation Pilots	N=33	119.9 ± 29.5	167.6 ± 58.2	47.8 ± 46.8	0.001

**Table 5:** Mean differences (95%-CI) in chair-rise test (seconds) and leg press strength (kg) effects after 12 weeks in the intervention groups between ProMuscle (PM) and ProMuscle in Practice (PiP), between PM and the ProMuscle Implementation Pilots (IP), and between IP and PiP

**Chair-rise test (sec)**	**Mean difference between two programs (95%-CI)**	**p-value**
PM - PiP	1.2 (-1.3; 3.6)	0.713
PM - IP	-1.3 (-4.3; 1.7)	0.854
IP - PiP	2.5 (0.1; 4.9)	0.035
**Leg press strength (kg)**	**Mean difference between two programs (95%-CI)**	**p-value**
PM - PiP	15.6 (-2.2; 33.4)	0.106
PM - IP	-15.1 (-35.2; 5.0)	0.209
IP - PiP	30.8 (14.3; 47.2)	<0.001

#### Chair-rise test

An improvement on chair-rise performance was found in the total study population as well as in each intervention group separately, with the highest increase in IP (-3.3 ± 4.2 s, p = 0.001). Figure 1 A and Table 5 show that the mean change in IP was significantly higher compared to the mean change in PiP.

#### Leg press strength

An improvement on leg press strength was found in the total study population and in each intervention group, with the largest change in IP (47.8 ± 46.8 kg, p < 0.001). Figure 1B and Table 5 show that the mean change in IP was significantly higher compared to the mean change in PiP.

### Contextual factors in ProMuscle, ProMuscle in Practice, and the ProMuscle Implementation Pilots

Contextual factors related to the PM, PiP and IP interventions are categorized according to relevant constructs of the CFIR domains: intervention characteristics (Table 6), inner setting and outer setting (Table 7), characteristics of individuals (Table 8) and process (Table 9). Under each table, similarities and differences between interventions are summarized.

**Table 6:** Intervention characteristics of the three interventions: ProMuscle, ProMuscle in Practice and the ProMuscle Implementation Pilots

**Intervention characteristics**			
An extensive description of the PM, PiP, and IP interventions was provided in Table 1 (exercise program) and Table 2 (nutrition program). Under this table, similarities and differences between the interventions were indicated separately for the exercise and the nutrition program.			
	**ProMuscle**	**ProMuscle in Practice**	**ProMuscle Implementation Pilots**
Adaptability	Exercise ▪ Strict guidelines implemented by researchers. Participants conducted training sessions according to protocol. Nutrition ▪ Participants received standard product for protein supplementation.	Exercise ▪ Most physiotherapists adhered to the training protocol, adjusting when necessary (intensity too high/too low). ▪ Participants and professionals indicated they would have liked more variation in type of exercises. ▪ Older adults could indicate their preference for a timeslot of the training sessions. Nutrition ▪ Number of consultations with dietitian was set. Dietitian provided individual advice, based on three-day food diary and preferences of participants, primarily including protein-rich products that were distributed for free. ▪ Participants mentioned they would like more variation in products, and that advice was hard to adhere to (25g per main meal). ▪ Consultations were scheduled together with participant.	Exercise ▪ Physiotherapists personalized the training intensity, based on the training protocol and adjusting when necessary (intensity too high/too low). ▪ Additional exercises were added based on capabilities of participants. ▪ Training sessions were scheduled considering daily activities of older adults. Nutrition ▪ Number of consultations with dietitian was personalized (minimum of three, more if needed). Dietitians provided individual advice, based on 24hr recall and preferences of participants, also included plant-based protein. Dietary protein intake around training sessions was emphasized in advice. ▪ Dietitian realized 25g protein per main meal might be hard to achieve and maintain on the long term, therefore she focused on 20-25g protein per main meal. ▪ Consultations were scheduled together with participant. ▪ Group-based meetings were scheduled prior to training sessions, to facilitate participation.
Complexity	▪ Duration of total intervention was 6 months. In this study, focus is on first 3 months. ▪ No implementation of intervention.	▪ Duration of total intervention was 6 months. In this study, focus is on first 3 months. ▪ Logistics at the start and during the program were sometimes constraining. Professionals were dependent on others for: receiving/repairing fitness machines and weights, receiving information on medical background and baseline food intake of participants, and provision of protein rich products. This led to delays, and some participants trained on a lowered intensity because of this.	▪ Duration of total intervention was 3 months; dietitian indicated this as a manageable period for participants. ▪ Professionals are project leader of their own intervention implementation, and therefore less dependent on others.
Cost	▪ Research was subsidized. ▪ Older adults could participate in the program for free. ▪ Researchers could conduct the program within their regular working hours.	▪ Research was subsidized. ▪ Older adults could participate in the program for free (first 12 weeks). ▪ Most professionals could conduct the program within their regular working hours.	▪ Research was not subsidized. ▪ Older adults could participate in the program for 70 euros per month (for training sessions with physiotherapist). Professionals indicated that this might lead to a selected group of participants, who can afford it. ▪ Consultations with the dietitian were reimbursed from the basic health insurance. ▪ Professionals could conduct the program within their regular working hours, and could cover the costs of the program using the participation contribution (physiotherapists) or the reimbursement from the health insurance (dietitians).

**Table 7:** The outer and inner setting of the three interventions: ProMuscle, ProMuscle in Practice and the ProMuscle Implementation Pilots

**Outer setting**			
	**ProMuscle**	**ProMuscle in Practice**	**ProMuscle Implementation Pilots**
External collaborations (cosmopolitanism) and policies	▪ No collaboration with external parties.	▪ Collaboration with care facilities led to involvement of professionals and availability of local facilities (i.e., training rooms). ▪ Health care professionals in the intensive support intervention were not always aware of the content of the moderate support program, limiting the transition.	▪ Collaboration with GP and medical practice assistant of GP facilitated recruitment and screening of participants. ▪ Collaboration with municipality facilitated subsidy for one group of participants. ▪ Collaboration with municipal health service led to the establishment of an implementation network overarching both municipalities.
**Inner setting**			
	**ProMuscle**	**ProMuscle in Practice**	**ProMuscle Implementation Pilots**
Structural characteristics of organization	▪ Intervention was conducted in the university (controlled setting).	▪ Intervention was conducted in care institution, within the municipality where participants live (real-life setting).	▪ Intervention was conducted in physiotherapist practices and dietitian practices within the municipality where participants live (real-life setting of the implementation pilots).
Networks & communications	▪ Researchers conducted the intervention completely; no further relevant communications in the inner setting.	▪ Overall, communication *among* physiotherapists or *among* dietitians went well. ▪ Communication *between* physiotherapists and dietitians could be improved in some cases.	▪ Communication among physiotherapists or *among* dietitians went well. ▪ Communications *between* physiotherapists and dietitians went well. ▪ Communication *between* physiotherapists and dietitians of different practices was facilitated during project meetings and included sharing experiences and best practices.
Implementation climate	▪ The study was performed to investigate efficacy of the intervention, and implementation was not part of the study aims.	▪ Most of the professionals chose to be involved in the intervention and enjoyed facilitating it. ▪ Most of the professionals received enough working hours to conduct the intervention, although some dietitians experienced too little time to conduct the program.	▪ Professionals chose to be involved in the intervention and enjoyed facilitating it. ▪ Conducting the program fell within their regular working hours.
Readiness for implementation	▪ The study was performed to investigate efficacy of the intervention, and implementation was not part of the study aims.	▪ Conducting the program fitted regular working procedures of professionals. However, the intervention was implemented in secondary care, while it would fit better within primary care or public health, based on the target population and professionals involved. ▪ In some cases, the training room was not suitable (noisy, not clean, small), and issues with training machines occurred (delay in delivery and repairments). ▪ Professionals could use materials such as guidelines, training protocols, calendars (protein intake) and registration lists. ▪ Professionals indicated they received participants’ baseline data and medical information too late from researchers, preventing them from tailoring the training intensity at the start of the program or causing a delay in providing the dietary advice.	▪ Conducting the program fitted regular working procedures of professionals. ▪ Available facilities included a spacious, safe environment for training sessions with training machines, and a room for consultations with the dietitian. ▪ Professionals could use materials such as information brochures, recruitment flyers, guidelines, training protocols, registration lists, and workshop materials for the nutrition course. ▪ In one of the municipalities a network with the medical practice assistant of GP was created to facilitate continuous recruitment of participants.

**Table 8:** Characteristics of individuals of the three interventions: ProMuscle, ProMuscle in Practice and the ProMuscle Implementation Pilots

Characteristics of individuals			
	ProMuscle	ProMuscle in Practice	ProMuscle Implementation Pilots
Professionals	▪ Researchers were skilled, committed, and motivated to conduct the intervention. ▪ Professionals conducted the program for the first time.	▪ Professionals had knowledge on the main components of the program, were experienced in working with the target group, and were motivated to conduct the program. ▪ Professionals conducted the program for the first time.	▪ Professionals were familiar with the content of the program, were experienced in working with the target group, and were motivated to conduct the program. ▪ Most of the professionals had already conducted the program several times and believed in the working mechanism of the program.
Participants	▪ Participants joined the program voluntarily and were motivated to participate in the program. ▪ The social aspect of the program was important to participants.	▪ Participants joined voluntarily and were motivated to participate in the program. ▪ The social aspect of the program was important to participants, and in some cases, participants continued the training sessions after the intervention (with the same group of participants).	▪ Participants joined voluntarily and were motivated to participate in the program. ▪ Professionals indicated that some participants lacked knowledge regarding the goal of the program. ▪ Social interactions among older adults highly stimulated participants to adhere to the program and in some cases to continue the training sessions after 12 weeks (with the same group of participants).

**Table 9:** Process of the three interventions: ProMuscle, ProMuscle in Practice and the ProMuscle Implementation Pilots

Process			
	ProMuscle	ProMuscle in Practice	ProMuscle Implementation Pilots
Planning and executing	▪ Researchers created a protocol and conducted the intervention according to it.	▪ Researchers created a protocol for conducting the intervention, based on the protocol of ProMuscle. ▪ Researchers trained professionals to conduct the intervention. ▪ Professionals adhered to the guidelines and adjusted the training intensity when necessary (too high/too low).	▪ Professionals could use the protocol of ProMuscle in Practice as an inspiration for conducting the intervention. ▪ Researchers trained professionals to conduct the intervention. ▪ Researchers discussed the protocol for implementing the intervention with professionals and discussed how this could be applied and adjusted to their specific setting. ▪ Basic elements of the intervention remained central, but there was room for own insights and adjustments according to available facilities/ resources and capabilities of individuals.
Engaging	▪ Researchers conducted the intervention themselves (i.e., providing training sessions).	▪ Managers of care organizations chose to be involved in the project and looked for physiotherapists and dietitians within their organization who were willing to practically conduct the intervention.	▪ Physiotherapists chose to be involved in the project and conducted the intervention themselves or instructed a colleague who was willing to be involved. ▪ Physiotherapists recruited dietitians to conduct the nutrition program of the intervention. ▪ A medical practice assistant of GP was involved to facilitate recruitment of participants.

### Intervention characteristics

An extensive description of the exercise and nutrition program for each intervention separately can be found in Tables 1 and 2*.*

#### Similarities

##### *Exercise program*

All interventions conducted RE training based on a training protocol two times per week, used training machines, and focused on the main muscle groups.

##### Nutrition program

All interventions focused on increasing protein intake during the main meals.

#### Differences

##### *Exercise program*

Physiotherapists in IP included individual intake consultations, whereas the other two interventions did not.

##### *Nutrition program*

During PM, protein supplemented beverages were provided for free. During PiP, a range of protein-rich products were provided for free. During IP, no food products were provided.

##### *Adaptability*

Training sessions in the PM program were conducted according to strict guidelines, whereas the training intensity was adjusted when necessary in PiP and IP. The latter program also offered additional exercises based on capabilities of participants. PM included no nutritional consultations. PiP included two individual consultations with a dietitian and offered optional additional phone contact. IP included a combination of group-based meetings and individual consultations with a dietitian and offered optional additional individual consultations.

##### *Complexity*

During PM, researchers conducted the intervention in a controlled setting and were not dependent on collaborations with external parties. In PiP, professionals were dependent on external parties for receiving materials and baseline data, causing delays in the training schedule of some participants. In IP, professionals are project leader of the intervention implementation, and therefore less dependent on others.

##### *Cost*

In PM and PiP, older adults could participate for free, whereas in IP, participants had to pay a monthly fee. In PM and IP professionals could conduct the program within their regular working hours, whereas some professionals in PiP could not (also due to the temporary character of the project).

### Outer and inner setting

#### Similarities

##### Inner setting:

*Readiness for implementation:* Professionals who conducted the intervention had access to materials such as guidelines and training protocols.

#### Differences

##### Outer setting: External collaborations and policies

There were no external collaborations during PM. During PiP, researchers collaborated with the municipal health service, sport facilities, care facilities and health care professionals. During IP, professionals collaborated with the GP and medical practice assistant of GP, municipalities, the municipal health service, and professionals of other physiotherapist practices.

##### Inner setting: Structural characteristics of organization

PM was conducted in the university, PiP in a care institution, and IP in physiotherapist and dietitian practices.

##### Inner setting: Networks & communications

There were no relevant communications during the PM program. During PiP, communications between physiotherapists and dietitians could have been improved in some cases. During IP, communications between physiotherapists and dietitians went well.

##### Inner setting: Implementation climate

Implementation was not a goal of PM. During the PiP program, some dietitians experienced too little time to conduct the program. During IP, conducting the program fell within regular working hours of professionals.

##### Inner setting: Readiness for implementation

During the PiP program, some training rooms were not suitable (noisy, not clean, or small) and issues with training machines occurred. During IP, a spacious and safe training room with training machines was available. During the PiP program, some professionals received participant’s baseline and medical data too late, causing delays or restrictions in the intervention. During IP, professionals conducted baseline measurements themselves and received medical data directly from the GP or the participants.

### Characteristics of individuals

#### Similarities

##### Professionals

Researchers or professionals involved were skilled and motivated to conduct the intervention.

##### Participants

Participants joined the program voluntarily and were motivated.

#### Differences

##### Professionals

Researchers or professionals involved in PM and PiP, conducted the intervention for the first time. Most of the professionals involved in IP were experienced in conducting the intervention, as they already conducted the program several times.

##### Participants

Professionals of PM, PiP and especially of IP indicated that the social aspect of the training sessions was very important to participants.

### Process

#### Similarities

##### Planning and executing

There was a protocol available for conducting the intervention.

#### Differences

##### Planning and executing

During PM, researchers conducted the intervention strictly according to the protocol. During PiP, professionals adhered to the protocol and adjusted the training intensity when necessary. During IP, basic elements of the protocol remained central, but professionals adjusted the intervention according to their facilities and to the capabilities of individuals.

##### Engaging

During PM, researchers conducted the intervention themselves. During PiP, managers of care organizations chose to be involved in the project and looked for physiotherapists and dietitians within their organization to conduct the intervention. During IP, physiotherapists chose to be involved in the project and conducted the intervention themselves or instructed a colleague who was willing to be involved. Physiotherapists involved a dietitian to conduct the nutrition program.

## Discussion

Effects on chair-rise test and leg press strength were not only found in the controlled setting but remained present in the real-life setting and were found to be even more pronounced in the real-life setting of the implementation pilots. The fact that effects vary across settings can be explained by several aspects, including the room for adapting and tailoring the intervention (*Intervention characteristics - adaptability*), the availability of and access to facilities (*Inner setting - readiness for implementation*), the involvement of experienced and independent professionals (*Characteristics of individuals - professionals*), and specific characteristics of the participants (*Characteristics of Individuals - participants*).

First of all, the experiences from a decade of working on the ProMuscle interventions were used to continuously develop and refine the intervention. It should be noted that the interventions we included in the current study are three successive rather than three separate interventions. The intervention was translated from the controlled to the practice setting. Continuous evaluation, in cooperation with professionals and participants involved, facilitated ongoing development of the intervention [11, 19]. Although effects often fade away when implementing an intervention in the real-life setting [13, 20], effects of the ProMuscle interventions remained present after both translation steps. This indicates that the development and translation process of ProMuscle was successful. As the three interventions slightly differ from each other, few considerations regarding the comparability of the results need to be addressed. First, it is important to notice that instruction manuals for measurements were used in all three studies to ensure standardisation. Besides, part of the research team was involved in all three studies. In this way, a proper transfer between the studies took place, which contributed to maintaining the quality of the training sessions. In addition, it should be emphasized that the core elements are similar in the three interventions. Core elements include progressive resistance exercise, mainly targeting the leg muscles, training in groups, and increasing protein intake. The goal of using core elements is to shift from a strictly manual focused intervention to a more scalable and sustainable intervention that meets the needs of the client majority. The use of core elements allows professionals to use their own judgment in combination with broad guidelines in order to fit an intervention to the client [21]. It is inevitable to slightly adjust the content of the program when transferring from a controlled to a practice setting, in order to fit the context. This paper advocates continuous monitoring to be able to indicate the effects of small adjustments.

As highlighted before, an essential step in transferring a health intervention from the controlled setting to the practice setting is adaptation [22, 23]. Adaptation includes adapting the intervention to fit a specific population or setting, and adapting intervention delivery while retaining the basic components of the intervention [24]. Ideally, adaptation proceeds via co-creation, meaning that researchers collaborate with local stakeholders and use their input to adapt the intervention [22, 24]. Quantitative as well as qualitative studies reported improved program outcomes and better implementation if intervention providers made small adaptations to the program [22]. This is in line with our results, which show increased effects when the intervention was adapted by professionals to the real-life setting of the implementation pilots. This is due to the fact that providers such as health care professionals are familiar with their community and are therefore able to fit the intervention to the needs and preferences of the local community, also called tailoring [22]. *Adaptability* was present in PiP but more pronounced in IP, which may have contributed to the difference in effects between the interventions. In IP, individual intakes facilitated tailoring of the intervention to the needs and capabilities of participants. A variety of exercises was offered, and dietary advice included a broad range of protein-rich food products, which made the program appropriate for a diverse group of participants. Besides, physiotherapists and dietitians took the activities of participants into account in order to fit the training sessions and consultations into their agendas. Systematic reviews also highlight the importance of personalized modification. Tailoring the intervention to the needs and capabilities of participants appeared to be a key element for success in physical activity as well as dietary interventions [25–28]. Besides, convenient scheduling was indicated as an enabling factor for participating in an intervention [25]. The results are in line with the process evaluation of PiP, in which tailoring and more variety in the intervention were highlighted as important elements [19].

An important aspect regarding *characteristics of individuals (professionals)* is the involvement of experienced professionals in delivering the intervention. The two physiotherapists and dietitians that conducted IP were already involved in the PiP study and could be indicated as ‘first users’ or champions. Champions are individuals that are dedicated to support the implementation and are characterised by their perseverance and strong believe in the intervention [2, 28, 29]. The involvement of champions, who are committed to and experienced with the intervention is associated with the intervention’s success [15, 31]. An important aspect related to the *Inner setting* is *readiness for implementation*. A relevant part of this aspect is the availability of and access to resources [15, 20]. Whereas during PiP physical space was sometimes suboptimal and training machines and data of baseline measurements were delivered too late, professionals of IP had direct access to their own facilities, including a spacious and safe environment with their own training machines, and data of baseline measurements. As baseline measurements were used as a starting point for the training program, receiving the data too late caused some delays in the PiP program. Other studies also indicate factors such as the availability of facilities, a safe, accessible, and convenient physical environment, and the access to documentation as enabling factors for intervention implementation [15, 25]. The fact that experienced professionals conducted IP independently, using their own facilities, without large delays or constraints, contributed to the intervention success and may partly explain the larger effects in IP compared with PiP.

In addition, baseline *characteristics of individuals (participants)* played a role in explaining intervention effects. Whereas the PM study included a relatively low number of female participants (36%), this number was relatively higher in the PiP study (62%) and IP (80%). As was reported in the in-depth analyses of the PiP study, women benefited to a greater extent from the intervention than men did [32]. The higher effects in IP could partly be explained by its high number of female participants, compared with the other two interventions. When adjusting our model (GLM) for sex, the significant effect of intervention setting remained present for leg press effects but disappeared for chair-rise effects. This implies that sex can partly explain the differences in effects on chair-rise performance between the settings.

Although many studies have been tested for efficacy in a controlled setting, few have been implemented in practice or were scaled-up [23, 24]. This can be described as the know-do gap, which reflects the gap between what is known in research and what gets done in practice [23, 33]. It indicates the need for studying intervention implementation. Up till now, the intervention was picked up by innovators and early adopters, according to the diffusion of innovations model [34]. Since IP showed positive results, it is time to additionally reach the early and late majority. An important point of attention of implementation, which was also highlighted in IP, are the costs related to the intervention, since financial aspects are often a barrier in implementation [4]. To gain insight in such barriers, but also enablers of implementation, and investigate how to systematically implement and consequently scale-up the ProMuscle intervention in the real-life setting, we recently started with the ProMuscle Implementation study (PUMP-fit).

Several strengths and limitations should be pointed out. A major strength of the intervention is its social aspect. Although the ProMuscle interventions are aimed at improving older adults’ muscle health and physical functioning, a positive side-effect is the emergent of strong social connections, a feeling of togetherness, new friendships, and even new relationships. Besides, the social aspect of the group training sessions highly motivated participants to adhere to the intervention in all three interventions, but especially during IP. Another major strength is the gradual development of the ProMuscle program. Continuous evaluation and development led to an effective intervention which can be implemented by professionals in the real-life setting. Only intervention groups were included in this study. Normally, it would not be suitable to highlight the intervention arms of these three studies to compare its effects, since the studies were not designed to be compared to each other. However, because the basic elements of the interventions are similar and the interventions expanded on the previous version, it provided us the unique opportunity to conduct the current study. A limitation that should be indicated is the low number of interviews with professionals involved in PM and IP. Consequently, the aspects highlighted from the interviews might not be generalizable to other professionals. To cover the opinion of a broader group on aspects related to implementation, focus group discussions and interviews are currently being conducted with professionals.

In conclusion, although we expected effects to fade away when implementing the intervention in the practice setting, the opposite appeared to be true. Effects of the intervention appeared to be strongest in the real-life setting of the implementation pilots. Specific contextual factors contributed to explaining the different findings across settings. For an intervention to remain successful in a new setting, it is essential to continuously reassess, renew, and refine, while remaining the intervention’s basic elements. To make sure health promotion programs reach a wide population, future studies should focus on systematic and sustainable implementation of effective interventions in the real-life setting.

## Data Availability

The datasets generated for this study are available upon reasonable request to the corresponding author.
